# Perspectives and challenges in developing and implementing integrated dengue surveillance tools and technology in Thailand: a qualitative study

**DOI:** 10.1371/journal.pntd.0012387

**Published:** 2024-08-14

**Authors:** Chawarat Rotejanaprasert, Peerawich Armatrmontree, Peerut Chienwichai, Richard J. Maude

**Affiliations:** 1 Department of Tropical Hygiene, Faculty of Tropical Medicine, Mahidol University, Bangkok, Thailand; 2 Mahidol-Oxford Tropical Medicine Research Unit, Faculty of Tropical Medicine, Mahidol University, Bangkok, Thailand; 3 Chulabhorn Learning and Research Centre, Chulabhorn Royal Academy, Bangkok, Thailand; 4 Princess Srisavangavadhana College of Medicine, Chulabhorn Royal Academy, Bangkok, Thailand; 5 Centre for Tropical Medicine and Global Health, Nuffield Department of Medicine, University of Oxford, Oxford, United Kingdom; 6 The Open University, Milton Keynes, United Kingdom; Huazhong University of Science and Technology Tongji Medical College, CHINA

## Abstract

**Background:**

Dengue remains a persistent public health concern, especially in tropical and sub-tropical countries like Thailand. The development and utilization of quantitative tools and information technology show significant promise for enhancing public health policy decisions in integrated dengue control. However, the effective implementation of these tools faces multifaceted challenges and barriers that are relatively underexplored.

**Methods:**

This qualitative study employed in-depth interviews to gain a better understanding of the experiences and challenges of quantitative tool development and implementation with key stakeholders involved in dengue control in Thailand, using a phenomenological framework. A diverse range of participants, including public health workers and dengue control experts, participated in these interviews. The collected interview data were systematically managed and investigated using thematic analysis to extract meaningful insights.

**Results:**

The ability to collect dengue surveillance data and conduct ongoing analyses were contingent upon the availability of individuals possessing essential digital literacy and analytical skills, which were often in short supply. Furthermore, effective space-time early warning and precise data collection were hindered by the absence of user-friendly tools, efficient reporting systems, and complexities in data integration. Additionally, the study underscored the importance of the crucial role of community involvement and collaboration among organizations involved in integrated dengue surveillance, control and quantitative tool development.

**Conclusions:**

This study employed a qualitative approach to gain a deeper understanding of the contextual intricacies surrounding the development and implementation of quantitative tools, which, despite their potential for strengthening public health policy decisions in dengue control, remain relatively unexplored in the Thai context. The findings yield valuable insights and recommendations for the development and utilization of quantitative tools to support dengue control in Thailand. This information also has the potential to support use of such tools to exert impact beyond dengue to a broader spectrum of diseases.

## Author summary

This study investigated the persistent public health challenge posed by dengue in tropical nations, with a specific focus on Thailand. Through qualitative research, it examined the potential of quantitative tools and information technology in integrated dengue control. Interviews with stakeholders, including public health workers and experts, revealed significant challenges. For instance, there was a shortage of essential skills for data collection and analysis, hampering effective surveillance and intervention. Additionally, issues such as the lack of user-friendly tools and complexities in data integration were identified. The study highlighted the importance of community involvement and collaboration among organizations. Recommendations included addressing these barriers by enhancing digital literacy and providing user-friendly tools. Overall, the study provided valuable insights into the development and utilization of quantitative tools, not only for dengue control but also for tackling a broader range of diseases.

## 1. Introduction

Dengue is a major mosquito-borne disease, with an estimated global burden of 390 million annual infections, of which around 96 million present with clinical symptoms [1]. The virus is primarily transmitted by *Aedes* mosquitoes and is prevalent in tropical and sub-tropical regions. While vaccines have been developed, their efficacy is limited, necessitating pre-vaccination screening, and some have sparse safety data and are not widely accessible. Consequently, vector control remains the main focus of public health interventions to interrupt the infection cycle [2]. Timely and effective large-scale surveillance and interventions are needed to reduce the serious impacts of dengue epidemics on health, healthcare systems, and economies [3,4].

Dengue has a significant impact on public health, particularly in Southeast Asia, where Thailand has one of the highest burdens of infection worldwide [5]. With approximately 100,000 annual cases reported to the Thai Ministry of Public Health, it poses a substantial burden on the healthcare system and households [6]. Dengue is endemic in Thailand, leading to epidemics every few years, particularly during the rainy season from May to October [7]. These outbreaks strain public health infrastructure, emphasizing the need for timely surveillance and control measures [8,9]. Information technology and quantitative tools play a crucial role in formulating effective dengue prevention and surveillance plans in Thailand.

Information technology and quantitative tools are useful to inform public health policy decisions about dengue control [10,11]. Several models have been developed to understand the drivers of dengue transmission and apply them to disease surveillance and control efforts [8,12–15]. However, creating these tools is just the first step; their effective utilization is equally crucial. Without practical application, their potential remains untapped. Despite the numerous information technology and quantitative tools developed for dengue control, their adoption has been limited. Moreover, there has been insufficient understanding of the experiences, challenges, and barriers faced by stakeholders incorporated into the development of quantitative tools for them to empower policy formulation and enhance dengue control in the country.

To address this gap, qualitative research can be employed to explore the challenges and successes of quantitative tools in dengue control programs. For instance, in a qualitative study conducted in Bangkok, the challenges and successes of fumigation campaigns for dengue control were explored [6]. However, no qualitative study has been undertaken to tackle the gap between quantitative tool development and practical implementation in Thailand. Given the high dengue endemicity in Thailand, the need has intensified to unearth effective public health management strategies and approaches for controlling and preventing dengue epidemics. This requires addressing the gap in translating the development of quantitative tools into guiding the efficient use of the limited resources invested. Consequently, this study aimed to understand the challenges faced in the development and application of quantitative tools and information technology in dengue control activities within Thailand.

To achieve our objectives, we conducted a qualitative investigation aimed at comprehending the experiences, perspectives, and challenges associated with quantitative tools enhancing dengue control efforts across various administrative levels. To ensure a thorough understanding, we selected participants from four stakeholder groups: public health professionals, policymakers, researchers, and informaticians, chosen for their expertise and roles in dengue control. Additionally, we analyzed the essential components necessary for improving future quantitative tool development. The insights gained have the potential to guide the development and utilization of such tools, not only for dengue but also potentially for addressing related diseases or similar environments in other countries.

## 2. Methods

### Ethics approval and consent to participate

This study was approved by the Ethics Committee of the Faculty of Tropical Medicine, Mahidol University. The submission number was TMEC 21–074 and the number of ethical approval certificate was MUTM 2021-071-01. Verbal consent was obtained and recorded in the audio files during the interview.

### 2.1. Study design and participants

This study employed a qualitative research design, chosen for its ability to explore deeply into the experiences of participants, surpassing the scope of quantitative methods [16]. Such research is typically conducted to investigate the meanings and interpretations held by individuals, providing a suitable approach to comprehending people’s underlying motivations [17], aligning well with the aims of our study. Given the impediments presented by the COVID-19 outbreak in Thailand during our study period, including travel and contact restrictions, we hence adopted the approach of conducting online in-depth semi-structured interviews. The phenomenological framework, a qualitative approach aiming to illuminate the essence of phenomena as experienced by individuals [18,19], was selected for this study to comprehensively understand the experiences, perspectives, and challenges associated with quantitative tools enhancing dengue control efforts across various administrative levels. This approach was chosen as it allows for an in-depth exploration of participants’ experiences, providing valuable insights into the complexities of utilizing quantitative tools in dengue control.

To capture a wide array of perspectives on these challenges, we engaged participants from four distinct stakeholder categories based on their roles and extensive experience in utilizing dengue control and surveillance quantitative tools in Thailand.

1. Public health professionals (PH): This group, sourced from both provincial and national levels, actively engages in dengue control endeavors. Their roles included a spectrum of tasks, including mosquito spraying operations, executing public health initiatives, and coordinating community health activities.
2. Policymakers (PM): This group represented the national dengue control program and local authorities within the Department of Disease Control, Ministry of Public Health, policymakers are instrumental in crafting dengue surveillance and control policies and guidelines. They oversaw the implementation of these measures by regional and local public health workers.
3. Scientist or epidemiologist (SE): This was selected from epidemiologists and scientists with expertise in laboratory and population-based dengue research. Their responsibilities encompassed a broad spectrum of activities, ranging from conducting laboratory studies to investigating various facets of dengue transmission, entomology, fieldwork, pathogenesis, and control strategies.
4. Informatician (IN): This group comprised programmers, analysts, engineers, and data experts, who have made significant contributions to dengue research and associated control activities. Their key responsibilities involved designing and implementing data collection systems, analyzing and interpreting data, and developing software tools to support dengue surveillance and control efforts.

The sample size for this study was determined through the application of theoretical saturation, a point reached when no further novel information is obtained from subsequent data collection [20]. Our pre-specified sample size calculation was informed by previous studies conducted in similar settings. For instance, a qualitative study on dengue control in Thailand involved face-to-face, in-depth interviews with 10 designated district officers in the Bangkok healthcare office, utilizing open-ended questions [6]. In another study, individual face-to-face interviews were conducted with healthcare personnel in Malaysia to gather their perspectives on the governance of dengue prevention and control with point of saturation observed after 19 interviews [21]. Similarly, a study examining the functioning of the Brazilian Dengue surveillance system obtained qualitative insights through interviews with 17 experts, focusing on data collection and reporting processes [22]. In light of these precedents, we pre-determined a sample size of approximately 16 in-depth interviews, a minimum of 4 participants per participant group, for our present study.

### 2.2. Data collection and analysis

Given the qualitative nature of our study, we employed a purposive sampling method to initially select participants for in-depth interviews in each stakeholder category. Our collaborators in the research community suggested the initial participants for scientists and informaticians, while the dengue national program recommended the initial public health personnel and policymakers for the interviews. Subsequently, we utilized a snowball sampling technique to expand our participant pool. Invitations to participate were extended via letters or email communications. Data collection occurred between November 2021 and October 2022.Prior to the interviews, participants received a written study overview and assurance of confidentiality. Verbal consent was obtained and recorded in the audio files during the interview. Demographic information was collected solely to characterize the interviewees, with no solicitation of identifiable data. The semi-structured interviews were conducted using a predefined question guide, focusing on key topics aligned with our study’s objectives. These interviews were audio-recorded and spanned in duration from approximately 30 to 60 minutes.

The interviews were transcribed verbatim from the audio recordings in their original language (Thai). Thematic analysis was conducted within a phenomenological framework [23,24]. A chronological review of the transcripts was undertaken to identify major themes, employing an inductive approach to data interpretation. Subsequently, the original data was coded and organized into sections with corresponding headings and subheadings [25]. Responses from multiple participants within each theme were consolidated, and in cases of theme inconsistencies, data was realigned into alternative themes until an appropriate structure was established. Any emerging themes that emerged during data collection and analysis were allocated additional headings and subheadings.

Manual coding was performed by PA. Codes that emerged from the initial translated interviews formed the basis of the codebook used to assess subsequent translated transcripts. The initial coding process was expanded into focused coding, where the association between different initial codes was explored based on frequency, sequence, correspondence, and similarity. CR independently repeated this process iteratively for all transcripts based on the codebook. Subsequently, CR and PA discussed the focused coding choices in detail. The final deductive codes were then grouped into meaningful categories, and sub-themes were generated by blending several categories together under the study objectives.

To ensure robustness, interviews and data collection transpired continuously throughout the period of subject enrollment. Data saturation, indicating the point at which no novel information was discerned from subsequent interviews [20,26], was evaluated. The study team made the decision on whether to continue additional interviews at this juncture. All qualitative data were managed using Microsoft Excel version 2108 and ATLAS.ti version 9. The research team held regular meetings and discussions to incorporate peer review, ensuring consistency and cross-checking the generated categories based on the study objectives. Additionally, team members collaboratively evaluated the findings and conclusions. To further minimize bias in data interpretation, the collected information was also shared with participants for their review. The final results were translated into English, with verbatim examples employed to illustrate key aspects of the themes. To protect the anonymity of our participants, pseudonyms were assigned to each participant category in relation to the quotations provided for each interviewee.

## 3. Results

While our initial sample size determination aimed for 16 interviews, the diversity in experience among public health workers, influenced by their locations and duties, led us to recruit more participants than originally planned. We reached the point of saturation in this category after conducting 8 interviews, while for the other participant groups, we interviewed 4 participants each. Demographic information for the 20 total participants, including nine females and eleven males, is provided in Table 1. The majority of participants held graduate degrees and had over five years of experience in dengue research and control activities.

Through analysis of the data obtained in our interviews, we identified several key conceptual themes. These include:

1. Understanding the multifaceted dynamics of dengue transmission and control.
2. Enhancing dengue surveillance through operational insights and technological innovations.
3. Experiences and challenges in utilizing quantitative tools for dengue surveillance.
4. Recommendations for developing quantitative tools and designing information technology for dengue control.
5. Community participation and collaborative efforts in dengue surveillance and control.

Detailed results for each theme are presented below.

**Table 1:** Demographics of participants with respondent characteristics and interview durations.

Stakeholder	Number of interviewees	Gender	Level of education	Dengue control experience (years)	Duration of interview (minutes.seconds)
Scientist or epidemiologist (SE)	4	M	Doctoral	< 5	43.58
		F	Doctoral	11–15	54.53
		F	Doctoral	11–15	34.28
		M	Master’s	5–10	48.04
Informatician (IN)	4	M	Doctoral	11–15	58.26
		M	Master’s	11–15	33.57
		M	Master’s	11–15	26.22
		F	Doctoral	5–10	30.52
Public health personnel (PH)	8	M	Master’s	> 15	50.13
		F	Master’s	5–10	50.32
		M	Bachelor’s	11–15	60.04
		M	Bachelor’s	> 15	49.25
		M	Bachelor’s	>15	42.19
		F	Bachelor’s	>15	39.11
		F	Master’s	11–15	30.05
		F	Bachelor’s	<5	42.01
Policymaker (PM)	4	M	Master’s	>15	63.23
		F	Master’s	5–10	39.01
		F	Master’s	>15	36.04
		M	Bachelor’s	>15	30.46

### Theme 1: Understanding the multifaceted dynamics of dengue transmission and control

The first theme derived from our interview data presents the intricate ecology of dengue transmission, shedding light on the multifaceted factors influencing the spread of the disease. While the primary focus of our study revolves around dengue surveillance, exploring this theme provides essential insights into the complex transmission pathways and associated risk factors identified during our interviews. By acknowledging the complexity of dengue transmission, we recognize its significance in informing the development of quantitative tools for surveillance. Our qualitative exploration with various stakeholders illuminates the diverse factors contributing to dengue transmission and control, which can serve as valuable inputs for the future development of surveillance technologies. Comparing these findings may aid in addressing gaps and enhancing the effectiveness of quantitative and information tools for dengue surveillance in diverse settings.

According to interviews with disease control professionals and dengue researchers, the spread of dengue was the result of multiple factors, including human carriers, vectors, and environmental elements. Improper storage of household items was highlighted as a key contributor to disease transmission, as these items could become breeding sites for mosquitoes. Preventive measures such as maintaining cleanliness in households and the proper disposal of containers were emphasized as effective strategies for controlling disease transmission.

Participants noted that controlling dengue was challenging due to the diverse factors contributing to its transmission, particularly environmental factors like weather patterns. Fluctuating weather patterns, particularly prolonged rainy seasons, significantly impacted mosquito breeding sites, elevating the risk of transmission. To address this challenge, they recommended proactive measures by governments and health authorities in affected communities. These measures included draining stagnant water, applying insecticides, and introducing mosquito repellent, which could disrupt the transmission cycle of dengue and protect public health.

SE: “…There are many complex factors that contribute to increasing dengue infection rates every year. There are many dimensions to this complexity, such as improper storage of household items that can become breeding sites for mosquitoes … Therefore, controlling dengue fever is very difficult and challenging…”

Throughout the interviews, participants also raised social aspects of dengue control. One critical issue was population migration, recognized as an important factor influencing disease spread. Understanding the behavioral patterns of migrant workers was deemed essential for effective dengue control. However, challenges emerged due to the transient nature of these individuals who often resided in rented accommodations near their workplaces, making it difficult to implement preventive measures.

PH: “…Population migration is also important, particularly in areas with high numbers of foreign workers. These workers often live in rented houses near factories and can be difficult to reach with disease prevention efforts… Environmental improvement can be challenging as they may not prioritize eliminating mosquito breeding grounds. This can lead to severe outbreaks. Understanding these factors is crucial in analyzing and planning disease control…”

The interviews mentioned about a noticeable pattern of dengue outbreaks initiating in urban centers or larger districts before spreading to rural areas. Popular tourist destinations **were** also susceptible to dengue outbreaks, highlighting the widespread impact of population movement on disease dynamics. Additionally, the timing of school calendars was identified as a significant factor affecting dengue cases. In Bangkok, for instance, the timing of school openings and closures influenced the incidence of dengue cases among students, showing the intricate interplay of social factors in dengue control.

Children and adolescents were identified as particularly vulnerable due to their frequent gatherings and close proximity to one another in schools, increasing the likelihood of transmission. Given the high risk among this group, maintaining cleanliness in schools and public areas, implementing proper hygiene practices, and promoting personal protection **were** emphasized as key strategies to reduce the risk of infection. Education and awareness programs conducted by the government and health authorities were recommended to promote proper hygiene practices among students and the general public.

PM: “…Population movement and density are probably very important because the pattern of dengue outbreaks that we see in a particular province usually starts in the cities or large districts before spreading to rural areas…”

PH: “…The opening and closing of schools in Bangkok has a clear impact on the number of dengue fever cases. During periods of school closures, such as during the 2020 outbreak, there was a significant decrease in the number of sick students…”

PH: “… Children are considered a high-risk group because they have to go to school, stay together, and there may be carriers in schools that make infection easier. Factors within the student or 5–14 year age group may be related to other external factors that affect behavior at this age…”

### Theme 2: Enhancing dengue surveillance through operational insights and technological innovations

This theme explores the operational aspects and challenges encountered in dengue surveillance and control efforts, as revealed by stakeholders interviewed in our study. The interviews highlighted the pivotal role of Thailand’s disease surveillance system in monitoring and managing dengue outbreaks. The dengue control program’s prevention model introduced various measures, including mosquito control, waste management, and continuous public health initiatives, aimed at curtailing the disease’s spread. Collaboration between provincial public health departments, local health workers, and village volunteers was emphasized to ensure comprehensive mosquito control activities across various settings. Understanding these operational activities and challenges faced by public health professionals at different levels provides valuable insights for quantitative tool developers. By comprehending the experiences and perspectives of stakeholders involved in dengue surveillance, developers can tailor quantitative and information technologies to address specific needs and challenges in Thailand’s context.

One such example of software utilized for dengue operational activity is the TanRaBad software. Developed collaboratively by international organizations, it was designed for monitoring dengue outbreaks and gathering entomological index data. This includes conducting visual surveys of larval habitats as part of routine activities conducted by the Thai Department of Disease Control (DDC) [27]. The software enables prompt identification of any rise in vector density, with larval indices computed and utilized as parameters for vector control measures. To streamline the larval survey process, the DDC has implemented a mobile application called TanRabad-SURVEY, facilitating real-time data collection from larval surveys nationwide since 2016 [27,28]. The implementation of this application can be used during a survey, aligning with larval survey protocols established by the World Health Organization (WHO) and the DDC [27,29]. These technological innovations not only enhance the efficiency of dengue surveillance but also support decision-making processes for more effective vector control strategies.

However, according to insights gathered during the interviews, the effectiveness of the software was significantly influenced by the digital literacy of its users. Many individuals responsible for data collection, including village health volunteers and public health workers, encountered technological challenges. These individuals often belonged to an older demographic and had limited familiarity with digital tools, which impeded the software’s efficiency. Despite the well-conceived features of the software, its proper utilization remained imperative to ensure data accuracy for effective surveillance planning.

Given the challenges associated with collecting surveillance data, a critical aspect to achieve these goals involved conducting a rigorous analysis of disease trends and risk factors. This analysis formed the bedrock for shaping emergency response strategies. Public health professionals heavily relied on data, principles, and logical reasoning to scrutinize and control disease outbreaks. Therefore, the collection of precise and dependable data assumed a pivotal role, substantiating policymaking and catalyzing the realization of public health goals.

SE: “…When it comes to being an epidemiologist, we always rely on data, principles, and reasoning. If we have data, it can be beneficial for us and the community. One thing that epidemiologists do is to collect and store data, interpret data, and report on disease investigations. This is very important in policy making and achieving the goals that address the problems. Good data collection leads to good analysis and interpretation…”

During the study, early detection and notification, rapid implementation of disease control measures, and enhancing the readiness of healthcare personnel for disease management, in conjunction with the analysis of disease trends and risk factors, emerged as fundamental components for both emergency planning and response. The central government’s objective of reducing dengue incidence and mortality rates highlighted another significant challenge in dengue control—the precision and quality of data collected by local public health personnel. The iterative data collection processes often led to errors and inaccuracies, significantly impeding the effective formulation of disease control policies.

Furthermore, dengue control measures and resources are overseen and funded by a range of local organizations, which include not only governmental public health workers under the Ministry of Public Health but also local administrative bodies under other ministries. Collaborative efforts among these diverse organizations are vital for timely detection and intervention to halt disease transmission. However, the absence of harmonious collaboration among the different administrative levels responsible for dengue control and management poses a significant obstacle, leading to a fragmented and suboptimal approach to disease control.

PH: “…We receive policies from the central government, which they call the main goal of the country, that is, to reduce the number of patients, and the mortality rate is the indicator of performance…. The analysis of the situation shows that the area should be concerned and take some actions or conduct analysis to identify and address the risks or… However, it is difficult sometimes to collect data and they can be missing…”

PH: “…In terms of cooperation between organizations in disease control, I thought it might be problematic as the local health authority is primarily responsible for disease control. However, some work in public health may be carried out with other organizations, which may or may not be under their power. This could potentially lead to a lack of attention to public health problems if the local health administration does not have the necessary authority…”

### Theme 3: Experiences and challenges in utilizing quantitative tools for dengue surveillance

In addition to operational challenges, our study uncovered experiences and significant issues related to the development and deployment of quantitative tools for dengue control. Discussions centered on resource allocation and disease control planning, particularly focusing on the creation of complex analytical models for forecasting future dengue case numbers. These sophisticated models often struggled with the demand for extensive and highly detailed data to ensure accuracy and effectiveness.

Furthermore, several data collection tools faced difficulties in achieving integration due to the complexity of consolidating information from diverse sources and organizations. This integration challenge resulted in a dearth of actionable insights, hindering the data collection process and impeding overall tool development. Analyzing dengue surveillance data, which is inherently intricate, requires the incorporation and integration of data from numerous sources and dimensions. In Thailand, these disparate data sources remain dispersed across various organizations, rendering their aggregation and comprehensive data analysis difficult. Furthermore, the development of quantitative tools and information technology encountered obstacles rooted in a misalignment with the actual needs and expectations of stakeholders.

Moreover, interviewees shed light on the challenges faced by village health volunteers, a critical user group responsible for monitoring and reporting dengue cases. Many of these volunteers encountered difficulties in understanding how to navigate the data collection tool, eroding their confidence in its effective use due to its complexity. Additionally, a subset of volunteers faced accessibility issues, as they lacked the financial means to acquire smartphones capable of running the application.

PM: “…Currently, the government is attempting to develop quantitative tools to control dengue. However, the obstacle is the lack of collaboration between organizations to integrate knowledge from experts in different fields across organizations. This means that the development of the tools cannot be implemented in real-life situations, and the available data cannot be utilized to its fullest potential…”

SE: “…The pandemic tracking app is a great system and idea. If we talk about the system, it is a great idea to have real-time monitoring for larvae surveys. However, there are limitations to its data collection due to the age of the community volunteers who use it. This is something they are currently trying to address…”

Interviewees also emphasized a critical challenge concerning the deficiency in data management tools and the requisite analytical skills, resulting in suboptimal data analytics. Although training programs had been developed, the practical application of these analytical skills had yet to reach a level of effective implementation. A concern that emerged during the interviews was the shortage of individuals proficient in computational programming languages such as R and Python. The utilization of data and the ability to conduct ongoing analyses were contingent upon the availability of individuals possessing these essential analytical skills, which were often in short supply. The absence of this foundational infrastructure posed a significant obstacle to the widespread adoption of technology and quantitative tools at different levels in decision-making processes. Moreover, financial constraints further complicated matters, as they hindered the integration of advanced tools and technologies, including artificial intelligence, despite a strong desire within the sector to leverage these capabilities.

Regarding disease control, the prevailing approach heavily relied on disease surveillance reports as the primary source of analysis. While surveillance and control mechanisms were in operation, the persistent issue of reporting delays and timeliness remained unresolved. Timeliness was a key component of effective surveillance, with local disease control authorities heavily dependent on timely reports to facilitate efficient disease control. Additionally, concerns regarding data coverage came to the fore, potentially impacting the overall efficacy of surveillance efforts. Although data management tools were employed to compile weekly disease situation reports, the data used for tracking and investigation were primarily drawn from the surveillance report. This also raised concerns about data coverage, particularly concerning private hospitals, which may not have been fully engaged in the reporting process.

PH: “…Currently, the public health sector lacks data management skills. While we use Excel at a certain level, proficiency in data analytics is still lacking. Though plans are underway to provide training, the reality is that people with skills in R and Python are still rare. While the surveillance data are manageable, there are still few individuals who can handle more complex datasets… There are budgetary limitations, and even if we want to use AI, there are still many constraints. Nevertheless, we are doing our best…”

SE: “…Dengue data is mostly based on disease reports to control the disease, and the analysis of high-risk areas. While there is already surveillance and control in place, the issue of reporting delays and timeliness still needs to be addressed…”

PH: “… Disease tracking and investigation are conducted by extracting data from the surveillance system, which contains information on patients receiving treatment in both public and private hospitals. However, the coverage of private hospitals may be incomplete depending on their willingness to participate…”

### Theme 4: Recommendations for developing quantitative tools for dengue control

The interviews produced valuable recommendations with several pivotal considerations for the development of quantitative tools for dengue control activities. Foremost among these was the importance of involving experts from various relevant departments, fostering the integration of diverse knowledge and perspectives at the early stages. This interdisciplinary collaboration was deemed crucial for comprehensively designing effective tools to address the multifaceted challenges posed by dengue. Additionally, the input from end-users emerged as a critical factor in the tool development process. This user-centric approach was seen as fundamental for enhancing the practical utility of these tools in real-world dengue control efforts.

IN: “…In reality, technology has the potential to solve the problem of dengue and control its spread at all levels, from national to community. However, the challenge lies in whether technology will be suitable to address the issue or not. Therefore, it is crucial to foster collaboration between organizations to tackle the problem effectively…”

Furthermore, the interviews emphasized the need to address both spatial and temporal dimensions in dengue control planning. Such tools would play a crucial role in promptly predicting dengue incidence outbreaks, accommodating reporting delays, and offering a comprehensive overview of the disease landscape from the national level down to local granularity. Spatial identification would provide precise coordinates, facilitating targeted mosquito elimination efforts, ensuring not only timely but highly accurate interventions. To make these critical insights readily accessible and usable, participants proposed creating a user-friendly interface or dashboard. This platform would serve as an information hub, encompassing space-time disease dynamics, enabling comprehensive and precise situation assessments and preparedness evaluations across different locations.

Among these recommendations, early warning with spatial identification emerged as an important strategy in dengue control. This approach gained particular importance due to the nationwide prevalence of the disease and resource constraints. Analyzing the disease landscape and identifying hotspots with the highest caseloads would enable targeted interventions. Knowing where to deploy additional vector control measures such as insecticide fogging and breeding site reduction, and diagnostic testing kits to achieve maximum impact all relied on accurate predictions of expected case estimates. Therefore, the development and deployment of quantitative tools hold promise for facilitating resource allocation and strengthening the effective response to the fluctuating dengue threat.

PH: “…Spatial identification will help control dengue more efficiently because the disease is widespread throughout the country. Due to resource constraints and budget limitations, intensive operations may not be possible in every area… By using timely analysis of the situation and identifying high risk areas, we can focus our efforts on those areas to prevent spread to neighboring areas…”

PM: “…Controlling dengue fever using quantitative tools like IT modeling is very useful because the disease has a clear seasonal pattern. We know when it will spread, but we don’t know how severe the outbreak will be each year. This makes it difficult to prepare resources not just in the public health sector but also in the local community…”

The interviews underscored the importance of user-friendly data tools not only for macro-level dengue control planning but also at the local level, considering variations in technology skills among local public health officers. Recommendations included the development of mosquito surveillance devices capable of autonomously alerting residents in affected areas to reduce reliance on village health volunteers with varying skills. Participants also stressed the need to collect data in an easily adaptable format for non-technical personnel. This approach could enhance data coverage, provided more user-friendly tools become available.

In pursuit of sustainable solutions for data integration and computational modeling development, participants proposed the idea of making all satellite and remote sensing data openly accessible. This approach would democratize data access for stakeholders and researchers, promoting more effective collaboration and research initiatives to enhance surveillance tools. However, implementing this transition would necessitate a shift in how Thai organizations handle data, particularly those with ties to foreign agencies, to embrace the concept of free data access. Despite the challenges, this move towards integration and inter-organizational collaboration was considered essential for creating practical, real-time tools and improving dengue surveillance and response in Thailand.

IN: “… Data should be stored in a database format, but non-data science personnel tend to summarize the data they collect, which makes it difficult to use the information… However, with the availability of free software, this issue has improved significantly. The sustainable solution is to access data by fixing the system, such as releasing data on a free access cloud, which would require organizations in Thailand to adapt to a new system…”

### Theme 5: Community participation and collaborative efforts in dengue surveillance and control

In addition to discussing dengue surveillance and quantitative tool development, interview participants highlighted the significance of community participation and collaborative efforts in controlling dengue. They identified a challenge in dengue control related to the attitudes and behaviors of individuals and the community’s willingness to adopt disease control measures. Participants emphasized that successful dengue quantitative tool development and solutions require collaboration among agencies involved in local community, vector control, and environmental efforts. Despite the availability of resources, motivating individuals to proactively engage in control measures can be challenging. Therefore, community involvement was recognized as an essential component of effective disease control. To enhance community involvement, participants stressed the importance of raising public awareness about the severity of the disease and emphasizing the need for prompt preventive actions.

Community engagement is crucial to strengthen disease control efforts. Dengue is not just a governmental responsibility but it has become a shared public and community concern. Consequently, community participation is essential in disease control. The effective prevention of disease spread needs a transformation in people’s behaviors and attitudes, involving the significance of awareness campaigns, educational outreach, and community engagement. Thus, supported by information from the interviews, it is important to incorporate local contextual factors into the development of quantitative tools and modeling for dengue surveillance.

SE: “…Even with innovative solutions and comprehensive databases, controlling the spread of dengue fever is not possible without active community participation. In cities, mosquitoes are in close proximity to people, making it challenging to combat the disease. Dengue is highly contagious and the vector is extremely resilient, able to survive in dry conditions with eggs that can last up to a year. Its unique biology enables it to maintain infection, making it difficult to eradicate. Thus, the most effective solution is control, and community personnel play a critical role…”

Beyond the community, effective management and the development of tools for dengue surveillance rely on collaboration among organizations in both the public and private sectors. Sharing data is a critical component of surveillance and information systems. Without this collaboration, effective surveillance becomes more challenging. In addition, this requires not only government funding and infrastructure support for dengue prevention but also active participation from community organizations and individuals. These contributions should also be directed toward localized activities aimed at addressing specific challenges in each area. While local public health personnel play a crucial role, participants acknowledged that relying solely on them for disease control is insufficient. Dengue control is a multifaceted challenge, encompassing both public health and environmental management. Therefore, collaborative efforts are essential, as a comprehensive approach is required to achieve dengue control goals.

PH: “…The biggest challenge in disease control is community involvement, which can be divided into three things: people, money, and management. Money and resources can be obtained, but managing people to behave as desired is difficult. We need good behavior, environmental improvements, and mosquito control, which are difficult because the public do not play their part…”

PH: “…If village health volunteers were required to do this in every village, it would not work. If the problem is not addressed properly from the beginning, it cannot be successfully resolved. Do you think it is an environmental problem or a people problem?…”

## Discussion

This study aimed to gain a deeper understanding of the experiences and challenges related to the development and application of quantitative tools in dengue control programs in Thailand. During the study, various aspects of surveillance activities and the use of quantitative tools in dengue control in Thailand were explored.

### Complexity of dengue transmission and vector surveillance

The study revealed the intricate dynamics of dengue ecology and its transmission pathways. Participants underscored a multitude of factors fueling dengue spread, notably the absence of specific treatments for dengue fever and the limited efficacy of existing vaccines. In light of these challenges, vector surveillance and management emerged as pivotal strategies for dengue prevention and control [30]. However, it was noted that traditional larval mosquito index monitoring may not consistently address dengue risk. Surveillance methods focusing on pupal and adult mosquito stages could offer more accurate estimates of dengue transmission risk, although implementation poses challenges [31]. Thus, the understanding of these multifaceted factors underscores the importance of comprehensive data collection, particularly in the context of vector control initiatives. Furthermore, the insights gleaned from this study regarding the complexity of dengue transmission hold significant implications for the future development of quantitative tools for dengue surveillance.

### Engaging communities for effective dengue control

Community participation emerged as a crucial aspect of dengue control efforts in our study, encompassing elements such as health literacy, self-protection practices, and proper household item storage. These factors were identified as significant contributors to disease transmission. This finding resonates with existing research, which has demonstrated that successful dengue vector control initiatives rely heavily on active community involvement [32,33]. Moreover, studies have identified common barriers to community engagement, including low awareness levels and a lack of government commitment and financial support, as observed in regions such as Vietnam and Cuba [34,35]. These examples underscore the complex interplay of factors influencing community participation in dengue control and highlight the necessity of community-driven approaches. Such approaches are pertinent not only to Thailand but also to other regions facing similar challenges. The effectiveness of dengue surveillance and the development of quantitative tools may be hindered by individual attitudes and community engagement barriers. Therefore, integrating local community factors into tool development and modeling processes can enhance the effectiveness of dengue surveillance strategies.

### Prioritizing user-centric approaches in quantitative tool development

This study identified a significant challenge related to the development of dengue surveillance tools, wherein these tools often prove impractical and fail to adequately address stakeholder requirements. Similar challenges have been observed in the development of healthcare tools and system processes, where stakeholders are frequently overlooked during the design phase. This oversight leads to the creation of products that remain underutilized, as they neglect the user’s context, needs, and inherent vulnerabilities within these systems [36,37].

To overcome this challenge, the adoption of user-centric methodologies, such as Design Thinking, is essential. These methodologies guide investigators in incorporating user needs and feedback throughout the development process [38,39]. Research has demonstrated the benefits of stakeholder involvement in addressing critical challenges within national health information systems, as demonstrated during the 2014 Ebola outbreak [40]. Closing the gap between research production and its real-world application remains a significant challenge for the health research system [41]. By involving stakeholders in the development of quantitative tools, their practicality and effectiveness are enhanced, thereby contributing to bridging this gap.

### Incorporating spatial and temporal dimensions in dengue modeling and control

While numerous dengue models employing various methods were proposed [42], some studies reported inaccuracies in dengue case predictions. These inaccuracies were attributed to the geo-spatial variations in climate and environment within regions [43]. Our study echoed similar findings, emphasizing the importance of developments that considered both spatial and temporal dimensions to effectively control dengue transmission. This emphasis aligned not only with research in Thailand but also in other regions [44,45]. Furthermore, the significance of addressing reporting delays and ensuring timely responses in controlling the spread of dengue transmission was underscored. This aligned with other modeling research conducted in Thailand [8,46,47]. Delays in reporting dengue cases frequently impeded timely interventions, highlighting the necessity of a system that ensured prompt reporting for early outbreak detection and more efficient resource management [48].

### Enhancing technology literacy and accessibility for dengue control tools

The study highlighted the significance of technology literacy and accessibility in the development of tools to support dengue control. Participants emphasized the importance of user-friendliness and effective data management to address these issues. Recognizing the technical limitations faced by many local public health workers, ensuring technological accessibility is pivotal for enhancing the usability of the developed quantitative tools. These findings align with previous research, which identified technology literacy as a potential barrier to implementation for health [49]. Additionally, other studies have indicated the positive impact of user interface design in health information systems on health worker performance [50,51].

### Study limitations

While our study findings offer valuable guidance for future tool development, it is important to acknowledge certain study limitations. Due to COVID-19 restrictions, our qualitative approach was limited to online in-depth interviews during the study period. From our experience in this study, we recognized that online interviews require more than facilitating the content and flow of the discussion. Engaging in online interactions on a research topic, particularly with unfamiliar individuals, proved mentally demanding. Additionally, we encountered occasional technical issues that caused lags in conversations during some interviews. However, we managed this challenge by adjusting the pace of the conversation with slightly longer pauses between sentences or questions, which helped maintain momentum. Nonetheless, online interviews enabled us to reach a wider range of participants, as the location of the research team no longer limited the geographic parameters of the study population. Online platforms have the potential to eliminate geographic barriers and may prompt researchers to approach their research questions differently. While online methods allow for broader sampling and recruitment, researchers should remain mindful of methodological concerns.

Due to purposive and snowball sampling, we recognize that the results may not comprehensively represent the views of the entire population [52,53]. Nevertheless, it is crucial to emphasize that our study provides an invaluable and contextually-rich understanding of the meanings and experiences associated with the development and implementation of quantitative tools and information technology for dengue surveillance in the Thai context. This nuanced insight holds significant value for future development efforts, particularly when addressing issues in Thailand that have been relatively less explored. It is also essential to exercise caution when attempting to apply these findings to other diseases or countries which have different surveillance systems, sets of interventions, and contributing factors, introducing uncertainty regarding the generalizability of our conclusions. Nonetheless, certain aspects of our findings may offer valuable insights, particularly for mosquito-borne diseases, with potentially broader applications.

## Conclusions

Dengue remains a significant public health challenge in tropical and sub-tropical regions, particularly in Thailand. While the potential of quantitative tools to inform and enhance public health policy decisions for dengue control is evident, the path to effective implementation is riddled with numerous challenges and barriers. This study has illuminated essential components crucial for strengthening the effectiveness of future quantitative tool development in the domain of dengue surveillance in Thailand. Key dimensions highlighted in our research include the importance of stakeholder engagement, capacity building, and the establishment of more robust data collection and sharing mechanisms. By addressing these factors, we can enhance the utility and impact of quantitative tools in supporting dengue prevention and control strategies.

Looking ahead, future research efforts could explore innovative approaches to overcome the challenges identified in this study. This could involve further investigation into user-centered design methodologies and the development of tailored interventions to address specific needs and barriers encountered by stakeholders. Moreover, increasing technological accessibility by ensuring new tools are user-friendly and providing necessary support and resources to all users, regardless of their technical proficiency, is essential. Additionally, investing in capacity building by offering training and resources to local health workers and organizations is crucial for effectively using and maintaining new surveillance technologies, ensuring long-term sustainability. he findings of this study have broader implications beyond Thailand, extending to other regions facing similar challenges in dengue surveillance and control efforts. By sharing our insights, we hope to contribute to the ongoing global efforts to combat vector-borne diseases and advance public health initiatives worldwide.
